# Molecular Characterization of *Anaplasma* spp. in Cattle from Kazakhstan

**DOI:** 10.3390/pathogens13100894

**Published:** 2024-10-12

**Authors:** Madina Kadyrova, Alexandr Ostrovskii, Kassym Mukanov, Amirkhan Kassen, Elena Shevtsova, Maxat Berdikulov, Gilles Vergnaud, Alexandr Shevtsov

**Affiliations:** 1 Laboratory of Applied Genetics, National Center for Biotechnology, Astana 010000, Kazakhstan; kadyrova@biocenter.kz (M.K.); ostrovskii@biocenter.kz (A.O.); mukanov@biocenter.kz (K.M.); kassen@biocenter.kz (A.K.); eleneshe@mail.ru (E.S.); 2 National Veterinary Reference Center, 150 let Abaya Street 22/3, Astana 010000, Kazakhstan; berdikulov.ma@mail.ru; 3 Institute for Integrative Biology of the Cell (I2BC), Université Paris-Saclay, CEA, CNRS, 91198 Gif-sur-Yvette, France; gilles.vergnaud@universite-paris-saclay.fr

**Keywords:** anaplasmosis of cattle, *A. centrale*, *A. marginale*, *A. ovis*, Kazakhstan

## Abstract

Bovine anaplasmosis is an infectious vector-borne disease caused by bacteria of the genus *Anaplasma*, which have a wide global distribution and represent a high economic burden for agriculture. The use of molecular genetic techniques has increased our knowledge of the species diversity of *Anaplasma* spp. and naturally susceptible animals. Monitoring studies allow us to assess the level of infection in herds, as well as the involvement of natural vectors in the processes of maintaining and spreading infection. Despite the high prevalence of *Theileria* and *Babesia* in cattle in Kazakhstan, there is no information on the distribution and species diversity of *Anaplasma* spp in this country. As part of this work, 7027 DNA samples isolated from the whole blood of cattle from 175 settlements in all 17 Kazakhstan regions were PCR-tested for the presence of *Anaplasma* spp. *Anaplasma* carriers were found in 1.3% (90 out of 7027) of the tested animals in 9 of the 17 regions of Kazakhstan. The highest percentage of infected animals was recorded in Turkistan (South Kazakhstan) and North Kazakhstan with 4.46% and 2.48% positive samples, respectively. The partial sequencing of *16S rRNA* and the *groEL* gene allowed us to identify five species of *Anaplasma*: *A. centrale*, *A. marginale*, *Candidatus Anaplasma* Mongolica, *A. ovis*, and Unknown *Anaplasma* with infection rates of 0.63%, 0.44%, 0.13%, 0.01%, and 0.01%, respectively.

## 1. Introduction

The genus *Anaplasma* (family *Anaplasmataceae*, order *Rickettsiales*) includes obligate intracellular alphaproteobacteria that reproduce in membrane-bound vacuoles and are transmitted to vertebrate hosts by ixodic mites. Since the last reclassification of *Anaplasmataceae* 20 years ago, two new species of *Anaplasma* have been identified. To date, the *Anaplasma* genus includes eight species, *A. phagocytophilum*, *A. marginale*, *A. centrale*, *A. ovis*, *A. bovis*, *A. platys*, *A. odocoilei,* and *A. capra,* and a large number of unclassified genovariants that cannot be assigned to known species [1]. *Anaplasma* species have a global distribution and cause anaplasmosis, a disease with a high negative impact in veterinary and public health [2].

The species *Anaplasma phagocytophilum* is the most significant in terms of public health. It is most common in the northern hemisphere and causes granulocytic anaplasmosis in humans, horses, and dogs. It is the cause of “tick-borne fever” (TBF) in domestic ruminants [3]. *Anaplasma marginale* is the dominant cause of anaplasmosis in cattle and other ruminants, and is found in tropical and subtropical regions of the world, including South and Central America, as well as in the United States (USA), southern Europe, Africa, Asia, and Australia [4]. It is more dangerous in animals over two years of age, especially those imported from non-endemic regions. The disease is clinically manifested by anorexia, jaundice, abortion, weight loss, reduced meat and milk production, and possibly death [5]. The species *A. centrale* infects various species of domestic and wild ruminants [6,7], but does not cause severe infection. Other *Anaplasma* species including *A. platys, A. bovis, and Anaplasma* sp. ‘Omatjenne’ have also been reported to infect cattle [8,9,10]. *Anaplasma platys* is a pathogen that primarily affects dogs. In cattle, the disease often proceeds subclinically, presenting with thrombocytopenia [11]. *Anaplasma bovis,* first discovered in Brazil, is also widespread in Africa, Asia, the Americas, and southern Europe. It affects cattle, buffaloes, sheep, goats, dogs, cats, and small mammals, with reported cases of infection in monkeys and humans as well [12]. In cattle, the infection may cause fever, decreased productivity, seizures, anemia, weight loss, and enlarged lymph nodes [13]. *Anaplasma* sp. ‘Omatjenne’ was first detected in healthy Boer goats in southern Africa, and was subsequently identified in cattle and buffalo in Africa and the Mediterranean Basin, but the clinical impact of this pathogen is still unclear [14].

Economic losses to livestock associated with *Anaplasma* spp. infection, the increasing incidence of human infections, and the discovery of new types of pathogens underscore the necessity of understanding the epidemiological situation. Diagnostic studies are essential for comprehending the epidemiology of anaplasmosis. However, serological methods present several limitations in the diagnosis of anaplasmosis, including the absence of antibodies in the early stages of the disease and low specificity due to cross-serological reactions among *Anaplasma* species [15]. Light microscopy is a simple and low-cost laboratory test with, however, limited applicability for animals that are chronic carriers of pathogens with low bacterial counts, which can lead to false negative results. Additionally, these tests are not very effective for leukocytic species such as *A. platys*, which is associated with thrombocytopenia, and for granulocytic species like *A. phagocytophilum* [16]. Molecular studies provide more accurate and reliable results and are based on the detection of gene markers such as *16S rRNA, groEL, gltA*, and major surface protein (*msp*) genes. For PCR screening, multi-copy *msp* genes are preferred, while for the identification of *Anaplasma* species based on nucleotide sequences, *rrs,* and *groEL* genes are currently considered the best choices [17].

Despite the prevalence of tick-borne infectious diseases in cattle in Kazakhstan [18], our knowledge on the spread of anaplasmosis is currently limited to information obtained from the light microscopy observation of blood smears in southeastern Kazakhstan [19]. The aim of the present study was to assess the distribution and species diversity of *Anaplasma* spp. throughout Kazakhstan in order to provide a baseline of *Anaplasma* spp. presence in Kazakhstan for future epidemiological surveillance.

## 2. Materials and Methods

### 2.1. Ethical Approval

This study was approved by the Ethics Committee of the National Center for Biotechnology (Protocol No. 2 dated 4 April 2022). Cattle owners confirmed their consent to blood sampling.

### 2.2. Collecting Samples

**Figure 1:**
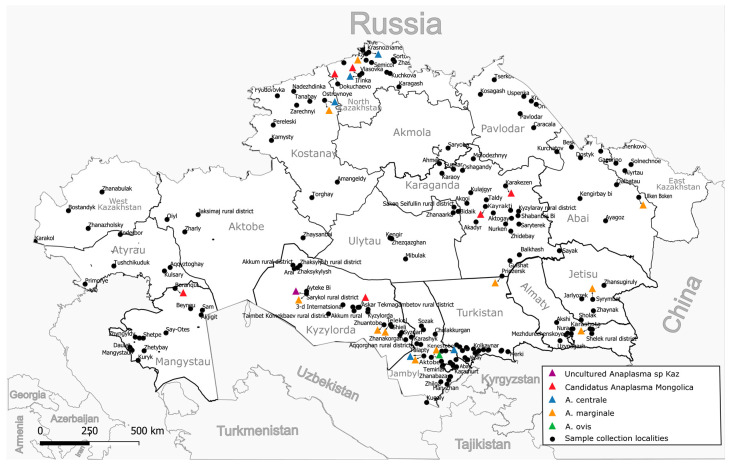
Sampling locations and distribution by identified species.

A total of 7027 whole blood samples were collected from cattle (cows) over three years of age from all 17 regions of Kazakhstan, 95 districts, and 175 settlements (Figure 1). The studied animals were kept in household farms and grazed in common herds of the respective settlement. The grazing of bulls is prohibited in common herds; therefore, the sample is limited to cows only. As a rule, the grazing of animals starts from 9 months of age and later; therefore, the inclusion of animals more than three years old guaranteed that the animals were on pasture for at least two grazing seasons from March to October (which may vary depending on the region). More detailed information about the blood collection season for the animals is provided in Supplementary Table S1. Blood was collected from the animals in July–August 2022 and 2023 using vacuum blood collection systems with di-potassium EDTA. Samples were transported to the laboratory within 48 h at 4 °C to 8 °C and were immediately used for DNA extraction.

### 2.3. DNA Isolation

Three hundred µL of blood were mixed with 900 µL of RBC buffer (Red Blood Cell Lysis Buffer, 150 mM NH_4_Cl (PanReac AppliChem, Darmstadt, Germany), 10 mM NaHCO_3_ (Thermo Fisher Scientific, Fair Lawn, NJ, USA), 1 mM EDTA (BioRad, Richmond, VA, USA), H_2_O). The mixtures were incubated for 5 min and centrifuged at 12,200× *g* for 5 min. The pellet was resuspended in 100 µL of buffer solution (400 mM NaCl (Titan Biotech Ltd., Rajasthan, India), 10 mM Tris-HCl pH 8.0 (BioRad, Richmond, VA, USA), 2 mM EDTA). Forty µL of 20% SDS (Sigma-Aldrich, Darmstadt, Germany) and 10 µL of 20 mg/mL Proteinase K (Magen, Guangzhou, China) were added and incubated at 50 °C for 2 h. A total of 500 µL of lysing solution was added (50 mm EDTA, 3.2 mM GuSCN (PanReac AppliChem, Darmstadt, Germany), 20 mM Tris-HCl (pH 7.4), 30% isopropanol (Sigma-Aldrich, St. Louis, MO, USA), 4% Triton X100 (Amresco, Solon, OH, USA), and the solution was stirred and incubated for 10 min at 60 °C. Seventy µL of sorbent (Silicon dioxide with a particle size of 0.5–10 µm (Sigma-Aldrich, St. Louis, MO, USA, S5631), prepared as described by R. Boom et al. [20], was added. The samples were incubated for 5 min at 60 °C, vortexing twice. They were kept at room temperature for 5 min and centrifuged at 600× *g* for 1 min. The precipitate was washed with 300 µL of buffer containing guanidine (3.2 M GuaSCN, 0.1 M Tris-HCl) and twice with 500 µL of 75% ethanol (75% ethanol (DOSFARM, Almaty, Kazakhstan), 10 mM Tris-HCl), each time carefully breaking up the sorbent. After each wash, the sorbent was pelleted by centrifugation at 1600× *g* for 1 min. Finally, the sorbent was dried at 60 °C for 3 min and DNA was eluted in 100 µL TE buffer (pH 8.0; 10 mM Tris, 1 mM EDTA, PanReac AppliChem, Darmstadt, Germany). DNA concentrations were measured using Nanodrop-1000 (Thermo Scientific, Wilmington, DE, USA).

### 2.4. Amplification of the groEL Gene Fragment

We designed new primers to amplify the *groEL* gene (Table 1). PCR amplification was performed in 25 µL reaction volume containing 12.5 µL BioMaster UDG HS-qPCR (2×) (Biolabmix, Novosibirsk, Russia), 1 µL (10 pmol/µL) of each primer, 5 µL DNA, and water up to 25 µL. Thermal cycling conditions using Mastercycler ProS (Eppendorf, Hamburg, Germany) were 2 min at 50 °C and 5 min at 95 °C initial denaturation, 35 cycles of 30 s at 95 °C, 40 s at 60 °C, and 50 s at 72 °C, followed by 5 min at 72 °C final elongation.

### 2.5. Nested Amplification 16S rRNA

Amplification of *16S rRNA* was performed by nested PCR using two pairs of primers (Table 1). Nested PCR was used to increase stage specificity, with primers specific to the genus *Anaplasma* spp. utilized at each stage. The PCR reaction was performed in a 25 µL reaction volume containing 12.5 µL BioMaster UDG HS-qPCR (2×), 1 µL (10 pmol/µL) of each primer for the appropriate round of amplification, and 5 µL DNA for round I PCR or 3 µL round I PCR product for round II PCR. Thermal cycling conditions using Mastercycler ProS (Eppendorf, Hamburg, Germany) were 2 min at 50 °C and 5 min at 95 °C initial denaturation, 30 cycles of 30 s at 95 °C, 40 s at 63 °C, 2 min for round I and 1.5 min for round II at 72 °C, followed by 5 min at 72 °C as final elongation.

### 2.6. Sequencing and Analysis

PCR products were run on a 1.5% agarose gel with ethidium bromide as the intercalating dye. PCR products were purified with magnetic particles, as described earlier [21]. Sanger sequencing was performed using the Big Dye Terminator v3.1 Cycle Sequencing Kit (Thermo Fisher Scientific, Vilnius, Lithuania) according to the manufacturer’s instructions. Sequencing reaction products were resolved on a GeneticAnalyzer 3730 xl (Applied Biosystems, Carlsbad, CA, USA). Sequences from the two strands were assembled using SeqMan (Lasergene, DNASTAR) [22]. Sequences were aligned using ClustalW. The phylogenetic analysis was conducted using the maximum likelihood method [23] and Tamura 3-parameter model with the discrete Gamma distribution with invariant sites with 5 rate categories. Bootstrap support was computed by comparing 100 replications. Trees were visualized using Mega 11 software v.11.0.13 [24]. MegAlignTM (Lasergene, DNASTAR) was used to determine the percentage of identity between sequences.

## 3. Results

### 3.1. Detection and Species Identification of Anaplasma spp. by the groEL Gene

The *groEL* gene fragment was amplified in 90 out of 7027 samples, which is 1.3% of the samples examined (Table 2). Animals with positive results were identified in 9 out of 17 regions of Kazakhstan, with the highest percentage of infected animals registered in the regions of Turkistan (South Kazakhstan) and North Kazakhstan. In total, 46 (4.36%) and 9 (2.48%) of the animals tested positive in these regions, respectively (Table 2). In the remaining seven regions, the percentage of infected animals ranged from 0.5 to 1.4%.

**Figure 2:**
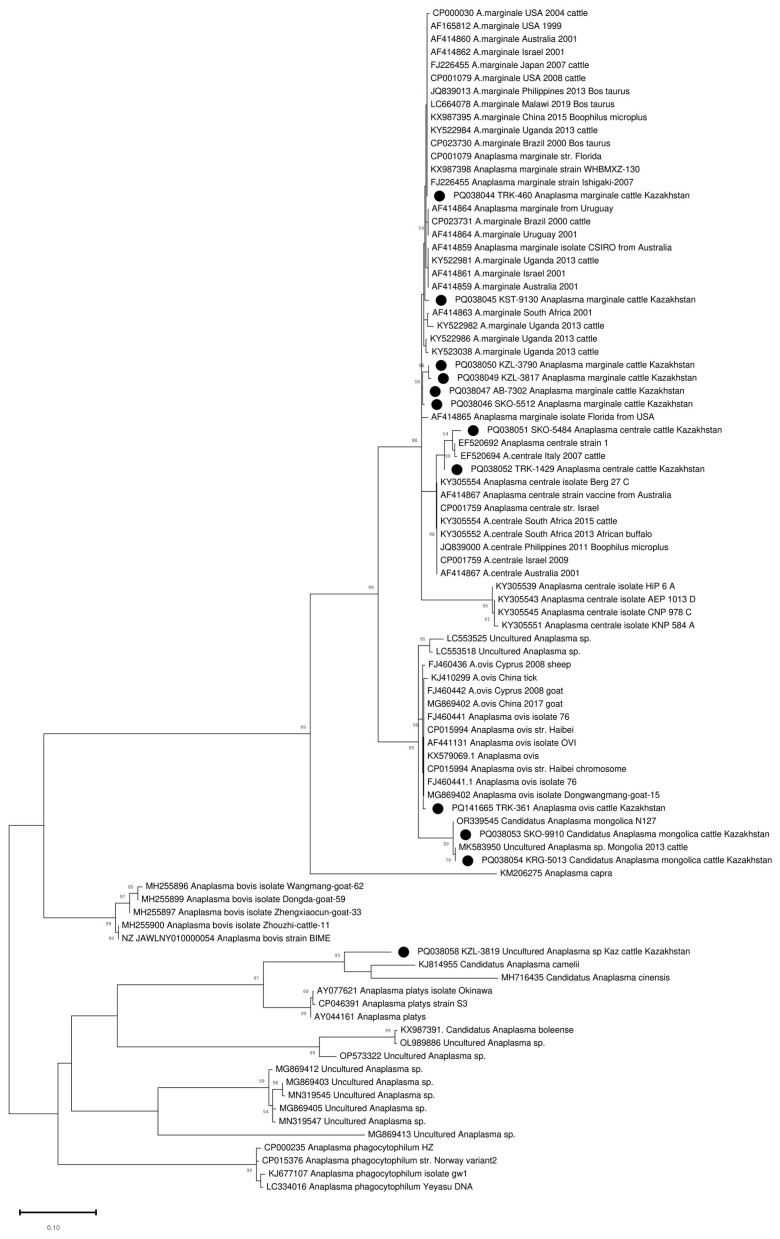
Phylogenetic tree based on the analysis of the partial sequence of the *groEL* gene. Only one sample of each genotype is included in the analysis; a complete analysis of the 86 sequences is shown in Figure S1. The sequences obtained in this study are labelled with ●.

The amplified *groEL* gene fragment of 401–404 bp (excluding primers) was sequenced in all 90 positive samples. A heterozygous signal was found in 5–10 positions in four samples. The remaining 86 samples were clustered into five clades (Figure S1). Thirty-one samples were clustered with *A. marginale* sequences (Figure S1, Table 2). These *A. marginale* positive samples originate from eight regions of Kazakhstan (Figure 1, Table 2). The average *A. marginale* infection rate of livestock in Kazakhstan was 0.44%. The maximum percentage of *A. marginale*-infected animals was registered in the Almaty region (3 out of 248 or 1.21%). In the other positive regions, the percentage of *A. marginale*-infected animals ranged from 0.4 to 0.85%. Kazakhstan’s *A. marginale* sequences define six genotypes (Figure 2). The partial *groEL* sequence from nine samples is identical to PQ038044, which is present on five continents (Figure 2 and Figure S1). The other five are unique.

Forty-four sequences clustered with *A. centrale* and formed two separate genotypes. One genotype combined eight sequences from two regions of northern Kazakhstan (North Kazakhstan and Kostanay regions). The second genotype included 36 *A. centrale* sequences identified in the whole blood samples of the animals from the Turkistan region (southern region of Kazakhstan) (Figure 2 and Figure S1). The average infection rate of *A. centrale* was 0.63%, and the maximum prevalence of *A. centrale* was recorded in the Turkistan region (3.42%). In the North Kazakhstan and Kostanay regions, *A. centrale* was detected in 1.38% and 0.7% of animals, respectively.

Nine sequences defined two closely related alleles differing at one nucleotide position and identical to two published sequences (OR339545 and MK583950), classified as *Candidatus Anaplasma* Mongolica (Figure S1). Samples from this cluster were collected in four non-contiguous regions, and the distance between collection sites varied from 104.43 to 1574.92 km. The average infection rate was 0.13%, and the infection rate in some regions was as high as 1%.

One sequence from the Turkistan region (South Kazakhstan) clustered with *A. ovis* sequences (Figure 2).

**Figure 3:**
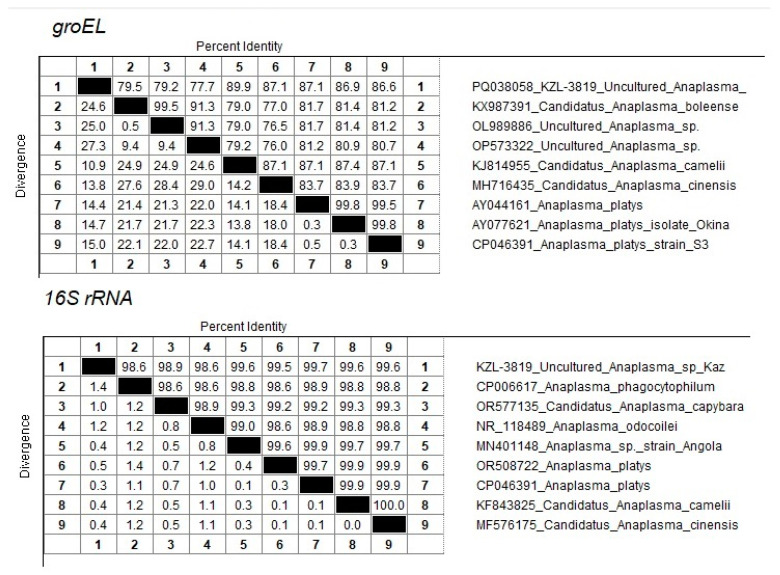
Percentage of sequence identity/divergence of KZL-3819 Uncultured *Anaplasma* sp Kaz accession PQ133430 with close neighbors.

One sequence (PQ038058 KZL-3819 Uncultured *Anaplasma* sp. Kaz) from the Kyzylorda region (South Kazakhstan) represents a separate branch in the clade together with close neighbors KJ814955 *Candidatus Anaplasma camelii*, MH716435 *Candidatus Anaplasma cinensis*, AY044161 *Anaplasma platys*, AY077621 *Anaplasma platys* isolate Okinawa, and CP046391 *Anaplasma platys* strain S3 (Figure 2). At the same time, the maximum percentage of identity of 89.9% was established with *Candidatus Anaplasma camelii* (Figure 3).

### 3.2. Identification of Anaplasma spp. by 16S rRNA

**Figure 4:**
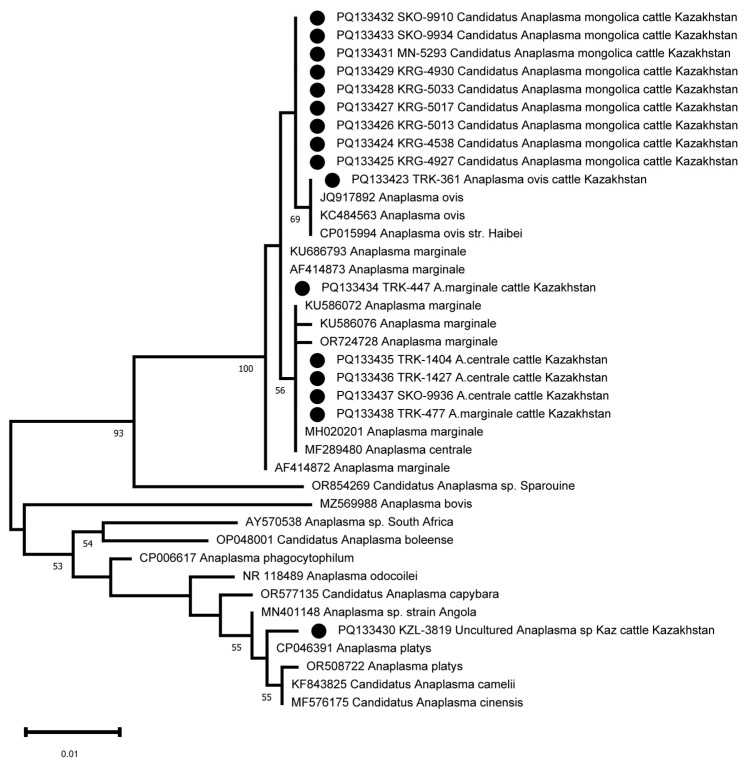
Phylogenetic tree based on the analysis of the *16S rRNA* fragment of 16 samples compared to published *Anaplasma* species 16S sequence data. The sequences obtained in this study are labelled with ●.

The amplification and sequencing of the *16S rRNA* fragment was used to clarify the taxonomic position of *Candidatus Anaplasma mongolica* and KZL-3819 Uncultured *Anaplasma* sp. Kaz. Sixteen samples were selected for 16S sequencing, including the nine samples identified as *Candidatus Anaplasma mongolica*, the *A. ovis* sample, and the KZL-3819 Uncultured *Anaplasma* sp Kaz sample. Three *A. centrale* and two *A. marginale* samples were included as controls. The primers developed were able to amplify the *16S rRNA* fragment in all 16 samples. The 16S sequences of the nine *Candidatus Anaplasma mongolica* samples are identical and differ at one position from the 16S sequence of the *A. ovis* sample and other public *A. ovis* 16S reference sequences (Figure 4). The five 16S sequences from the samples tentatively identified as *A. centrale* and *A. marginale* clustered as expected with public 16S sequences from *A. centrale* and *A. marginale* strains. The PQ133430 16S sequence from the KZL-3819 sample is located as a separate branch in a cluster with MN401148 *Anaplasma* sp. strain Angola, OR508722 *Anaplasma platys*, CP046391 *Anaplasma platys*, KF843825 *Candidatus Anaplasma camelii,* and MF576175 *Candidatus Anaplasma cinensis* (Figure 4). The closest neighbor of PQ133430 is CP046391 *Anaplasma platys*, which differs at two positions among 734 bps (99.7% sequence identity, Figure 3).

## 4. Discussion

Anaplasmosis is a vector-borne or mechanically transmitted disease caused by members of the genus *Anaplasma* of the order *Rickettsiales* [25]. Anaplasmosis causes significant damage to the livestock industry, with an estimated economic burden of USD 660 [26] per clinical case. Currently, there are no vaccines available to prevent the infection of livestock with anaplasmosis, but their use facilitates the clinical course of the disease [5]. In the absence of specific prevention, the focus is on the diagnosis, the culling of infected animals, and the exclusion of the import of infected animals into safe areas. Therefore, data on the distribution and species diversity of circulating *Anaplasma* spp. are needed. We conducted the first study in Kazakhstan to assess the spread of anaplasmosis in cattle using PCR targeting the *groEL* gene followed by the species identification of the pathogen through sequencing. As a result of this study, 1.3% (90 out of 7027 examined) of the animals were found to carry *Anaplasma*. The percentage of infected animals differed between regions, reaching a maximum of 4.46% in the southern region of Kazakhstan. Five types of *Anaplasma* were identified: *A. centrale, A. marginale, A. ovis, Candidatus anaplasma mongolica,* and Unknown *Anaplasma.*

Kazakhstan is located in the center of Eurasia, the republic’s area is 2724.9 thousand km^2^, and more than 80% of the country’s territory is occupied by dry steppes. The diversity of landscape and climatic conditions contributes to the existence of a wide variety of tick species, including more than 30 species of ticks belonging to the *Ixodidae* family, which are recognized as vectors of a number of tick-borne diseases [27]. Nevertheless, only four regions of southern Kazakhstan are recognized as disadvantaged by tick-borne diseases (TBDs) among cattle [18]. Much attention among TBDs in cattle is paid to the causative agent of teileriosis and babesiosis; there are few data on the spread of anaplasmosis in livestock in Kazakhstan. Our study detected the presence of *Anaplasma* spp. in cattle in 9 of the 17 regions of Kazakhstan. Importantly, we did not detect *Anaplasma* spp. in eight regions, suggesting that they might currently be free of *Anaplasma* spp. The southern regions of Kazakhstan are recognized as disadvantaged for blood-parasitic diseases; therefore, the detection of *Anaplasma*-infected animals in the Turkistan, Kyzylorda, Almaty, and Jetisu regions is to be expected. The only exception among southern regions is the Jambyl region, where the absence of *Anaplasma*-infected animals might be due to the geography. Samples for *Anaplasma* testing from this region were collected from localities situated in the mountains. It is known that the density of the main tick vectors is significantly lower in mountainous areas compared to steppe pastures. The detection of infected animals in the northern and northwestern regions of the country may indicate two independent pathways for the introduction of anaplasmosis into Kazakhstan. Most of the regions free of *Anaplasma spp.* are located in the interior of the country.

Of the 90 samples, 4 samples showed a clearly mixed profile, suggesting coinfection with more than one type of *Anaplasma.* Coinfection with various species of *Anaplasma* in domestic animals has previously been described in several studies. In Korea, coinfection with *A. bovis* and *A. phagocytophilum* reaches 16% [28], and in Algeria the coinfection of *A. marginale* and *A. centrale* was reported in 10% of cases [29]. The coinfection of various types of anaplasmas is of concern due to the possibility of recombination and the emergence of new variants that are dangerous to public health and livestock [30].

We showed here that *A. centrale is* currently the most common type of *Anaplasma* in cattle in Kazakhstan. It was detected in 44 of 7027 samples (0.63%). Infected animals were detected in southern (Turkistan region) and northern Kazakhstan (North Kazakhstan and Kostanay regions). The maximum percentage of infection (3.4%) was detected in the Turkistan region. The species *A. centrale* is found worldwide and can infect various species of domestic and wild ruminants [6,7]. The infection rate varies: in Tunisia and Algeria, *A. centrale* is detected in 15.1% and 39.4% of cattle. A rate of 14.4% and 18% was reported in Pakistan and Turkey, respectively [31,32]. In Chongqing province in southwestern China, *A. centrale* was detected in 7.83% of examined cattle, which was second only to *A. bovis*, reported in 8.41% of animals [33]. In Kyrgyzstan, the infection rate of *A. centrale* in cattle is 1.1% [34]. *Anaplasma centrale* causes mild infections in cattle, with the formation of immunity against *A. marginale*, which does not protect animals from infection, but excludes the severe course of infection [35]. Therefore, *Anaplasma centrale* is considered a naturally attenuated variant that has been used as a live vaccine for more than 100 years. It is currently widely used in South Africa, Israel, South America, and Australia [4]. In this regard, the high infection of vaccinated cattle with *A. centrale* is observed in these regions [36,37].

Despite the century-long history since the description of *A. centrale* by Arnold Theiler, the debate on the taxonomic position of the species continues [38]. An analysis of the *16S rRNA*, *groEL*, and *msp4* gene sequences, and the *Msp1a*/*Msp1aS* structure of *A. marginale* and *A. centrale* isolates from South Africa, groups *A. centrale* into a separate clade from *A. marginale*, which, with a combination of morphological differences (*A. centrale* forms smaller and more central morulae in erythrocytes), allows *A. centrale* to be considered a separate species [39]. The inclusion of additional sequences of the *groEL* and *16S rRNA* genes in the phylogenetic analysis showed that the sequences of *A. marginale* and *A. centrale* are not clustered separately on the basis of both genes, requiring careful consideration of the taxonomic position of *A. centrale* [1]. In our study, *A. centrale* and *A. marginale* strains clustered separately, which is associated with the use of a fragment of the *groEL* gene c 171 of 574 nucleotides. Previously, Ben Said et al., by analyzing the complete sequence of the *groEL* gene among *Anaplasma* spp., found that two regions have a discriminating potential between *A. centrale* and *A. marginale* (region 1 between positions 1 and 546 and region 2 between positions 1059 and 1650) [40]. An interesting fact is the separate clustering of the *groEL* gene sequence of samples from southern and northern Kazakhstan, indicating independent introduction of the pathogen into these regions. The analysis of *16S rRNA* did not allow us to differentiate *A. centrale* and *A. marginale*, since only one nucleotide distinguishes these species at position 156 A/G [41], which has not been sequenced using the primers we have proposed.

*Anaplasma marginale* turned out to be the second most widespread species, but was identified in more areas. There is also high genetic diversity: seven genotypes were identified, and while only one genotype combining eight samples had genetic analogues in the NCBI database, the rest formed a separate cluster and were unique to Kazakhstan. Our study showed a low prevalence of *A. marginale* among cattle in Kazakhstan; the total infection was 0.44%, while the highest number of positive animals was detected in the Almaty region with 1.2%. We found only one study on *A. marginale* in Kazakhstan. In that study, 256 samples from cattle were examined by light microscopy and 48.9% were found to be infected with *A. marginale* [19]. In that study, the authors examined more than ten thousand animals, but there is no information about the criteria for selecting material and forming a sample for microscopic examination, which makes it difficult to assess the true percentage of infection. The infection rate of cattle in neighboring countries differs. In Kyrgyzstan, for example, a PCR study found that the infection rate with *A. marginale* was 11.6%, while in four out of five regions the pathogen was not detected [42]. In southern Xinjiang (China), bordering Kazakhstan, the infection rate of *Anaplasma* spp. varies from 3.3% to 12.8%, while *A. marginale* has not been identified [43,44]. In Russia, there is a significant difference in the distribution of *A. marginale* in the regions, ranging from 8.3 to 71.1% [45,46].

Nine animal carriers of *Candidatus Anaplasma mongolica* were identified in four regions. The regions are located in the southern, central, and northern parts of Kazakhstan at a distance of more than 1000 km. Previously, this species was identified in ticks in Inner Mongolia (China) [47] and in ticks and blood samples of cattle in Mongolia, where the infection rate of cattle with this species was 31.8% [48]. Our data indicate a wider distribution area of this type of *Anaplasma* in Asia. The lack of information on the pathogenicity and severity of the disease caused by this pathogen requires additional research and increased observations.

The species *A. ovis* was identified in one animal in the Turkistan region (southern Kazakhstan). *A. ovis* causes anaplasmosis in sheep and goats and is much more host-specific than *A. phagocytophilum* [49]. However, *A. ovis* cannot be considered a strictly species-specific pathogen, as the number of *A. ovis* detections in other species has increased recently, most likely due to the development of molecular genetic methods for the species identification of *Anaplasma* [50]. Infection with *A. ovis* has been confirmed in wild ungulates: European roe deer (*Capreolus capreolus*) [51], big horn sheep (*Ovis canadensis*) and mule deer (*Odocoileus hemionus*) [52], and red deer (*Cervus elaphus*) and spotted deer (*Cervus nippon*) [53]. The infection of reindeer (*Rangifer tarandus*) in Mongolia reaches 80% [54]. Among domestic animals, in addition to the main hosts, sheep and goats, *A. ovis* has been found in camels in Iran and Tunisia [55,56] and cattle in China and Mongolia [43,57]. This is the first study confirming the presence of *A. ovis* among cattle in Kazakhstan. The detection of *A. ovis* in atypical species of domestic animals is observed in regions with a high incidence of sheep anaplasmosis. For example, in Iran, more than 50% of herds and 28% of sheep are infected with *A. ovis* [58]; in Tunisia, the infection rate of small ruminants reaches 80% [59]; and in Mongolia, the average is 70% of infected animals [60]. According to our unpublished data, the detection rate of *A. ovis* in sheep in the Turkistan region is indeed high and amounts to 42%, in agreement with these previous reports.

An unidentified species of *Anaplasma* spp. was identified in an animal from the Kyzylorda region. It is genetically closest to *Candidatus Anaplasma camelii,* with 90% *groEL* gene identity and *99.7% 16S rRNA identity with A. platys*.

In general, our study indicates a low infection rate of cattle with anaplasmosis in Kazakhstan. Perhaps this is due to the fact that, for centuries, the territory of Kazakhstan was dominated by nomadic livestock breeding, where the main animal species were sheep, horses, and camels [61]. The transition to a sedentary lifestyle in the early 20th century increased the number of cattle [62].

## 5. Conclusions

This is the first study using molecular genetic methods to investigate the species diversity and prevalence of *Anaplasma* spp. in privately owned cattle in Kazakhstan. The results of this study showed a low level of infection of cattle with anaplasmosis, and a high species diversity of circulating *Anaplasma* spp. This view of the current situation of *Anaplasma* spp. will help to monitor the epidemiological situation of the infection, and hopefully help detect emerging trends sufficiently early to allow for the implementation of countermeasures such as vaccination and the anti-tick treatment of animals and pastures.

The limitation of our study is the lack of information on the clinical manifestation of anaplasmosis in Kazakhstan. Further studies on the health impact and species diversity of *Anaplasma* in cattle and ticks in Central Asia will help clarify the pathogenicity and range of *Candidatus Anaplasma mongolica* and uncharacterized *Anaplasma* species.

## Figures and Tables

**Table 1:** Primers used for amplification of *groEL* and *16S rRNA*.

Target Gene	Primer Name	Primer Sequences (5′–3′)	PCR Size (bp)
*groEL*	*groEL*_Anapl_all_F	aaggatggatayaaggtmatgaa	about 445
	*groEL*_Anapl_all_R	cgcggwcaaactgcatac	
*16S rRNA*, round I	16S_Anap_F150	atctacctagtagtatgggatagccact	about 1293
	16S_Anap_R1460	ctgcctccttacggttggcg	
*16S rRNA*, round II	16S_Anap_F397	agctatgccgcgtgagtgag	about 914
	16S_Anap_R1315	atgccctcgagttgcagagga	

**Table 2:** Results of PCR tests for *Anaplasma* spp. in cattle in the regions of Kazakhstan and their species identification.

Region	Number of Herds Studied	Total Samples Examined	Total Positive for PCR *groEL*	*A. centrale*	*A. marginale*	*A. ovis*	*Candidatus Anaplasma mongolica*	Unknown *Anaplasma* spp.	*Anaplasma* spp. Co-Infection
Turkistan	23	1054	46 (4.36%)	36 (3.42%)	9 (0.85%)	1 (0.09%)	0	0	0
North Kazakhstan	15	362	9 (2.48%)	5 (1.38%)	2 (0.55%)	0	2 (0.55%)	0	0
Kyzylorda	21	980	12 (1.22%)	0	10 (1.02%)	0	1 (0.10%)	1 (0.10%)	0
Karaganda	23	500	7(1.40%)	0	2 (0.40%)	0	5 (1.00%)	0	0
Kostanay	9	428	6 (1.40%)	3 (0.70%)	2 (0.47%)	0	0	0	1 (0.23)
Abai	8	400	4 (1.00%)	0	2 (0.50%)	0	0	0	2 (0.50%)
Mangystau	15	200	2 (1.00%)	0	0		1 (0.50%)		1 (0.50%)
Almaty	11	248	3 (1.21%)	0	3 (1.21%)	0	0	0	0
Jetisu	4	200	1 (0.50%)	0	1 (0.50%)	0	0	0	0
Jambyl	16	955	0	0	0	0	0	0	0
Ulytau	6	400	0	0	0	0	0	0	0
Pavlodar	7	300	0	0	0	0	0	0	0
West Kazakhstan	4	200	0	0	0	0	0	0	0
Aktobe	4	200	0	0	0	0	0	0	0
Atyrau	5	200	0	0	0	0	0	0	0
East Kazakhstan	3	200	0	0	0	0	0	0	0
Akmola	1	200	0	0	0	0	0	0	0
Total	175	7027	90 (1.3%)	44 (0.63%)	31 (0.44%)	1 (0.01%)	9 (0.13%)	1 (0.01%)	4 (0.06%)

## Data Availability

All data from this project are publicly available from NCBI, GenBank Ac#: PQ038044-PQ038058, PQ133423-PQ133438, PQ141665.

## Supplementary Materials

The following supporting information can be downloaded at: https://www.mdpi.com/article/10.3390/pathogens13100894/s1, Figure S1: A phylogenetic tree based on the analysis of a fragment of the nucleotide sequence of the *groEL* gene in 86 samples obtained in this study; Table S1: Characteristics of PCR-positive *Anaplasma* spp. samples.
