# Supplementary material for: Molecular Characterization of Anaplasma spp. in Cattle from Kazakhstan
Source: Pathogens. 2024 Oct 12;13(10):894. doi: 10.3390/pathogens13100894 (PMC11510537; doi:10.3390/pathogens13100894)
Supplement: Supplementary file 1 [file pathogens-13-00894-s001.zip › pathogens-3186415-Supplementary Table S1.pdf]

Table S1. Characteristics of PCR-positive Anaplasma spp. samples

No. of samples	Region	District	Locality	Latitude	Longitude	Date of blood sampling	Sex	groEL species assignment	Accession number
TRK-354	Turkistan	Otrar	Aktobe	42.799580	68.713191	July	Female	A. marginale	
TRK-361	Turkistan	Otrar	Aktobe	42.799581	68.713192	July	Female	A. ovis	PQ141665
TRK-368	Turkistan	Otrar	Aktobe	42.799582	68.713193	July	Female	A. marginale	
TRK-447	Turkistan	Otrar	Talapty	42.861180	68.294127	July	Female	A. marginale	
TRK-450	Turkistan	Otrar	Talapty	42.861181	68.294128	July	Female	A. marginale	
TRK-452	Turkistan	Otrar	Talapty	42.861182	68.294129	July	Female	A. marginale	
TRK-460	Turkistan	Otrar	Talapty	42.861183	68.294130	July	Female	A. marginale	PQ038044
TRK-469	Turkistan	Otrar	Talapty	42.861184	68.294131	July	Female	A. marginale	
TRK-476	Turkistan	Otrar	Talapty	42.861185	68.294132	July	Female	A. centrale	
TRK-477	Turkistan	Otrar	Talapty	42.861186	68.294133	July	Female	A. marginale	
TRK-480	Turkistan	Otrar	Talapty	42.861187	68.294134	July	Female	A. marginale	
TRK-1388	Turkistan	Baydibek	Kenestobe	43.036235	69.610353	August	Female	A. centrale	
TRK-1389	Turkistan	Baydibek	Kenestobe	43.036236	69.610354	August	Female	A. centrale	
TRK-1390	Turkistan	Baydibek	Kenestobe	43.036237	69.610355	August	Female	A. centrale	
TRK-1391	Turkistan	Baydibek	Kenestobe	43.036238	69.610356	August	Female	A. centrale	
TRK-1392	Turkistan	Baydibek	Kenestobe	43.036239	69.610357	August	Female	A. centrale	
TRK-1393	Turkistan	Baydibek	Kenestobe	43.036240	69.610358	August	Female	A. centrale	
TRK-1394	Turkistan	Baydibek	Kenestobe	43.036241	69.610359	August	Female	A. centrale	
TRK-1395	Turkistan	Baydibek	Kenestobe	43.036242	69.610360	August	Female	A. centrale	
TRK-1396	Turkistan	Baydibek	Kenestobe	43.036243	69.610361	August	Female	A. centrale	
TRK-1398	Turkistan	Baydibek	Kenestobe	43.036244	69.610362	August	Female	A. centrale	
TRK-1399	Turkistan	Baydibek	Kenestobe	43.036245	69.610363	August	Female	A. centrale	
TRK-1401	Turkistan	Baydibek	Kenestobe	43.036246	69.610364	August	Female	A. centrale	
TRK-1402	Turkistan	Baydibek	Kenestobe	43.036247	69.610365	August	Female	A. centrale	
TRK-1404	Turkistan	Baydibek	Kenestobe	43.036248	69.610366	August	Female	A. centrale	
TRK-1406	Turkistan	Baydibek	Kenestobe	43.036249	69.610367	August	Female	A. centrale	
TRK-1408	Turkistan	Baydibek	Kenestobe	43.036250	69.610368	August	Female	A. centrale	

TRK-1409	Turkistan	Baydibek	Kenestobe	43.036251	69.610369	August	Female	A. centrale	
TRK-1410	Turkistan	Baydibek	Kenestobe	43.036252	69.610370	August	Female	A. centrale	
TRK-1411	Turkistan	Baydibek	Kenestobe	43.036253	69.610371	August	Female	A. centrale	
TRK-1412	Turkistan	Baydibek	Kenestobe	43.036254	69.610372	August	Female	A. centrale	
TRK-1413	Turkistan	Baydibek	Kenestobe	43.036255	69.610373	August	Female	A. centrale	
TRK-1414	Turkistan	Baydibek	Kenestobe	43.036256	69.610374	August	Female	A. centrale	
TRK-1415	Turkistan	Baydibek	Kenestobe	43.036257	69.610375	August	Female	A. centrale	
TRK-1416	Turkistan	Baydibek	Kenestobe	43.036258	69.610376	August	Female	A. centrale	
TRK-1417	Turkistan	Baydibek	Kenestobe	43.036259	69.610377	August	Female	A. centrale	
TRK-1418	Turkistan	Baydibek	Kenestobe	43.036260	69.610378	August	Female	A. centrale	
TRK-1419	Turkistan	Baydibek	Kenestobe	43.036261	69.610379	August	Female	A. centrale	
TRK-1421	Turkistan	Baydibek	Kenestobe	43.036262	69.610380	August	Female	A. centrale	
TRK-1423	Turkistan	Baydibek	Kenestobe	43.036263	69.610381	August	Female	A. centrale	
TRK-1424	Turkistan	Baydibek	Kenestobe	43.036264	69.610382	August	Female	A. centrale	
TRK-1425	Turkistan	Baydibek	Kenestobe	43.036265	69.610383	August	Female	A. centrale	
TRK-1426	Turkistan	Baydibek	Kenestobe	43.036266	69.610384	August	Female	A. centrale	
TRK-1427	Turkistan	Baydibek	Kenestobe	43.036267	69.610385	August	Female	A. centrale	
TRK-1428	Turkistan	Baydibek	Kenestobe	43.036268	69.610386	August	Female	A. centrale	
TRK-1429	Turkistan	Baydibek	Kenestobe	43.036269	69.610387	August	Female	A. centrale	PQ038052
KZL-3780	Kyzylorda	Kazaly	Sarykol rural district	45.732557	62.115942	July	Female	A. marginale	
KZL-3790	Kyzylorda	Kazaly	Sarykol rural district	45.732558	62.115943	July	Female	A. marginale	PQ038050
KZL-3793	Kyzylorda	Kazaly	Sarykol rural district	45.732559	62.115944	July	Female	A. marginale	
KZL-3794	Kyzylorda	Kazaly	Sarykol rural district	45.732560	62.115945	July	Female	A. marginale	
KZL-3796	Kyzylorda	Kazaly	Sarykol rural district	45.732561	62.115946	July	Female	A. marginale	
KZL-3815	Kyzylorda	Kazaly	Sarykol rural district	45.732562	62.115947	July	Female	A. marginale	
KZL-3817	Kyzylorda	Kazaly	Sarykol rural district	45.732563	62.115948	July	Female	A. marginale	PQ038049
KZL-3819	Kyzylorda	Kazaly	Sarykol rural district	45.732564	62.115949	July	Female	Uncultured Anaplasma sp Kaz	PQ038058
KZL-3823	Kyzylorda	Kazaly	Sarykol rural district	45.732565	62.115950	July	Female	A. marginale	
KZL-4015	Kyzylorda	Shieli	Telekol rural district	44.259722	66.685556	August	Female	A. marginale	

KZL-4380	Kyzylorda	Shieli	Zhuantobe	44.267588	66.311218	August	Female	A. marginale	
KZL-4538	Kyzylorda	Syrdariya	Askar Tekmagambetov	45.057148	65.052480	August	Female	Candidatus Anaplasma mongolica	
KRG-4842	Karaganda	Priozersk	Priozersk	46.028969	73.708027	July	Female	A. marginale	
KRG-4855	Karaganda	Priozersk	Priozersk	46.028970	73.708028	July	Female	A. marginale	
KRG-4927	Karaganda	Shet	Kayrakty	48.849579	73.603831	July	Female	Candidatus Anaplasma mongolica	
KRG-4930	Karaganda	Shet	Kayrakty	48.849580	73.603832	July	Female	Candidatus Anaplasma mongolica	
KRG-5013	Karaganda	Karkaraly	Karakezen	49.595000	75.058333	August	Female	Candidatus Anaplasma mongolica	PQ038054
KRG-5017	Karaganda	Karkaraly	Karakezen	49.595001	75.058334	August	Female	Candidatus Anaplasma mongolica	
KRG-5033	Karaganda	Karkaraly	Karakezen	49.595002	75.058335	August	Female	Candidatus Anaplasma mongolica	
MN-5250	Mangystau	Mangystau	Zhyngyldy	44.198686	51.700201	July	Female	Anaplasma spp. co-Infection	
MN-5293	Mangystau	Beyneu	Boranqul	46.208008	54.469042	July	Female	Candidatus Anaplasma mongolica	
AB-7302	Abai	Kokpekti	Ulken Boken	48.781692	82.694252	July	Female	A. marginale	PQ038047
AB-7303	Abai	Kokpekti	Ulken Boken	48.781693	82.694253	July	Female	A. marginale	PQ038048
AB-7305	Abai	Kokpekti	Ulken Boken	48.781694	82.694254	July	Female	Anaplasma spp. co-Infection	
AB-7312	Abai	Kokpekti	Ulken Boken	48.781695	82.694255	July	Female	Anaplasma spp. co-Infection	
ALM-7593	Almaty	Enbekshikazakh	Karashota	43.649992	78.183065	July	Female	A. marginale	
ALM-7601	Almaty	Enbekshikazakh	Karashota	43.649993	78.183066	July	Female	A. marginale	
ALM-7607	Almaty	Enbekshikazakh	Karashota	43.649994	78.183067	July	Female	A. marginale	
JT-8504	Jetisu	Eskeldi	Syrymbet	44.856519	78.682484	August	Female	A. marginale	
KST-9129	Kostanay	Sarykol	Ostrovnoye	53.342672	65.477797	August	Female	Anaplasma spp. co-Infection	
KST-9130	Kostanay	Sarykol	Ostrovnoye	53.342673	65.477798	August	Female	A. marginale	PQ038045
KST-9132	Kostanay	Sarykol	Ostrovnoye	53.342674	65.477799	August	Female	A. centrale	
KST-9133	Kostanay	Sarykol	Ostrovnoye	53.342675	65.477800	August	Female	A. centrale	

KST-9136	Kostanay	Sarykol	Ostrovnoye	53.342676	65.477801	August	Female	A. centrale	
KST-9139	Kostanay	Sarykol	Ostrovnoye	53.342677	65.477802	August	Female	A. marginale	
SKO-5484	North Kazakhstan	Mamlyut	Krasnozname nnoe	54.977354	68.808946	July	Female	A. centrale	PQ038051
SKO-5485	North Kazakhstan	Mamlyut	Krasnozname nnoe	54.977355	68.808947	July	Female	A. centrale	
SKO-5490	North Kazakhstan	Mamlyut	Krasnozname nnoe	54.977356	68.808948	July	Female	A. centrale	
SKO-5503	North Kazakhstan	Mamlyut	Pokrovka	54.928831	68.636794	July	Female	A. marginale	
SKO-5512	North Kazakhstan	Mamlyut	Pokrovka	54.928832	68.636795	July	Female	A. marginale	PQ038046
SKO-9910	North Kazakhstan	Timiryazev	Dokuchaevo	53.914759	66.486964	July	Female	Candidatus Anaplasma mongolica	PQ038053
SKO-9934	North Kazakhstan	Esil	Il'inka	54.201824	68.010444	August	Female	Candidatus Anaplasma mongolica	
SKO-9936	North Kazakhstan	Esil	Il'inka	54.201825	68.010445	August	Female	A. centrale	
SKO-9942	North Kazakhstan	Esil	Il'inka	54.201826	68.010446	August	Female	A. centrale	
