# Supplementary figures and images for: Molecular Characterization of Anaplasma spp. in Cattle from Kazakhstan
Source: Pathogens. 2024 Oct 12;13(10):894. doi: 10.3390/pathogens13100894 (PMC11510537; doi:10.3390/pathogens13100894)

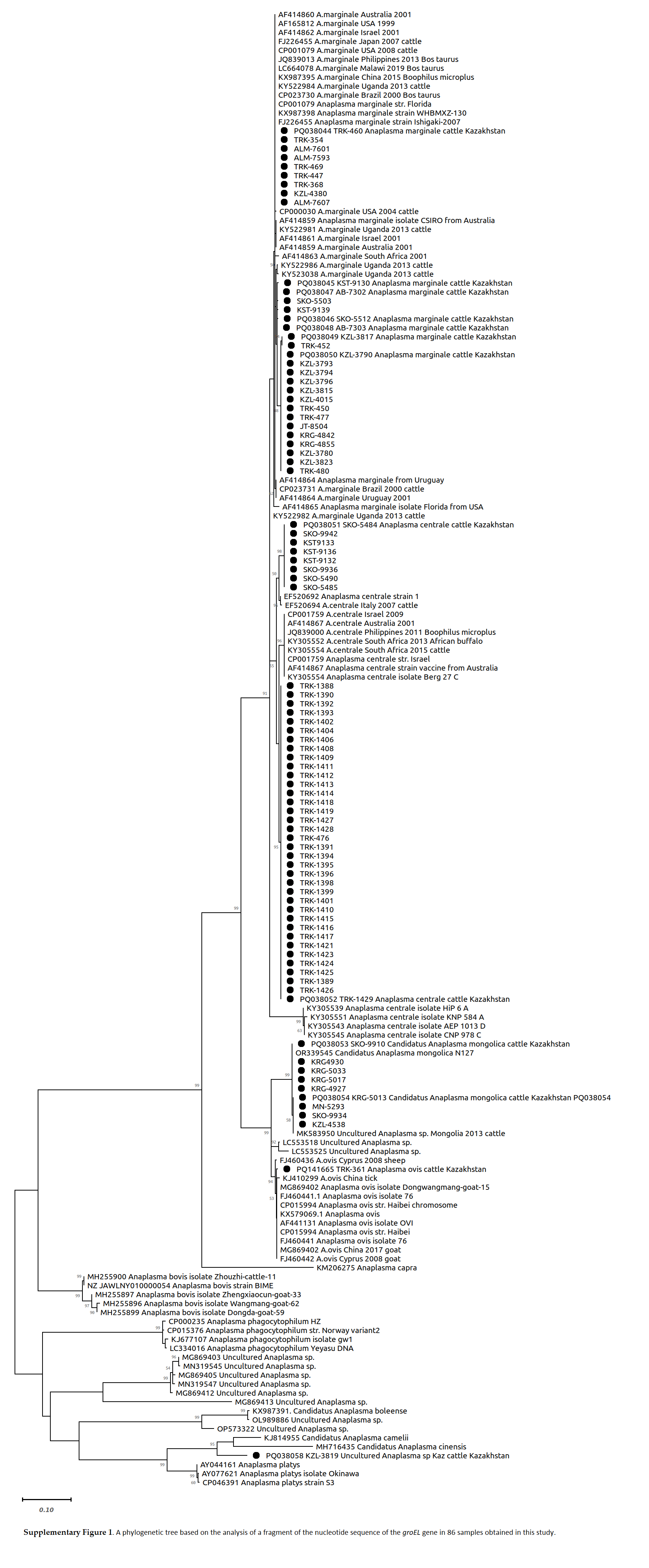

Supplement: Supplementary file 1 [file pathogens-13-00894-s001.zip › pathogens-3186415-Supplementary Figure S1.tif]
